# A Multimodal fNIRS–EEG Dataset for Unilateral Limb Motor Imagery

**DOI:** 10.1038/s41597-026-07807-x

**Published:** 2026-08-10

**Authors:** Lufeng Feng, Baomin Xu, Haoran Zhang, Bihai Lin, Zuxuan Deng, Sidi Tao, Chenyu Liu, Shifan Jia, Li Duan, Ziyu Jia

**Affiliations:** 1 Beijing Jiaotong University, Beijing, 100044 China; 2 Nanyang Technological University, 50 Nanyang Avenue, Singapore, Singapore; 3 Simon Fraser University, Vancouver V5A 1S6, Burnaby, Canada; 4 Institute of Automation, Chinese Academy of Sciences, Beijing, 100044 China

## Abstract

Unilateral limb motor imagery (MI) plays an important role in upper-limb motor rehabilitation and precise control of external devices, and places higher demands on spatial resolution. However, most existing public datasets focus on binary- or four-class left–right limb paradigms that mainly exploit coarse hemispheric lateralization, and there is still a lack of multimodal datasets that simultaneously record electroencephalography (EEG) and functional near-infrared spectroscopy (fNIRS) for unilateral multi-directional MI. To address this gap, we present MIND, a public motor imagery fNIRS–EEG dataset based on a four-class directional MI paradigm of the right upper limb. The dataset includes 64-channel EEG recordings (1000 Hz) and 51-channel fNIRS recordings (47.62 Hz) from 30 participants (12 females, 18 males; aged 19.0–25.0 years). We analyze the spatiotemporal characteristics of EEG spectral power and hemodynamic responses, and provide baseline classification summaries for EEG, fNIRS, and combined modalities as technical validation of task-related information in the dataset. We expect that this dataset will facilitate the evaluation and comparison of neuroimaging analysis and decoding methods.

## Background & Summary

Brain-computer interfaces (BCIs) are systems that acquire and decode brain activity signals and translate them into control commands for external devices^1^. According to the signal acquisition modality, BCIs can be divided into invasive and non-invasive types^2^. Motor imagery (MI) is one of the most important paradigms in non-invasive BCIs^3^. With the rapid expansion of BCIs into clinical practice, rehabilitation, and human-computer interaction, the potential application scenarios of MI have become increasingly diverse, including upper-limb functional reconstruction^4,5^, daily assistive control^6^ and continuous trajectory^7^ guidance.

**Fig. 1:**
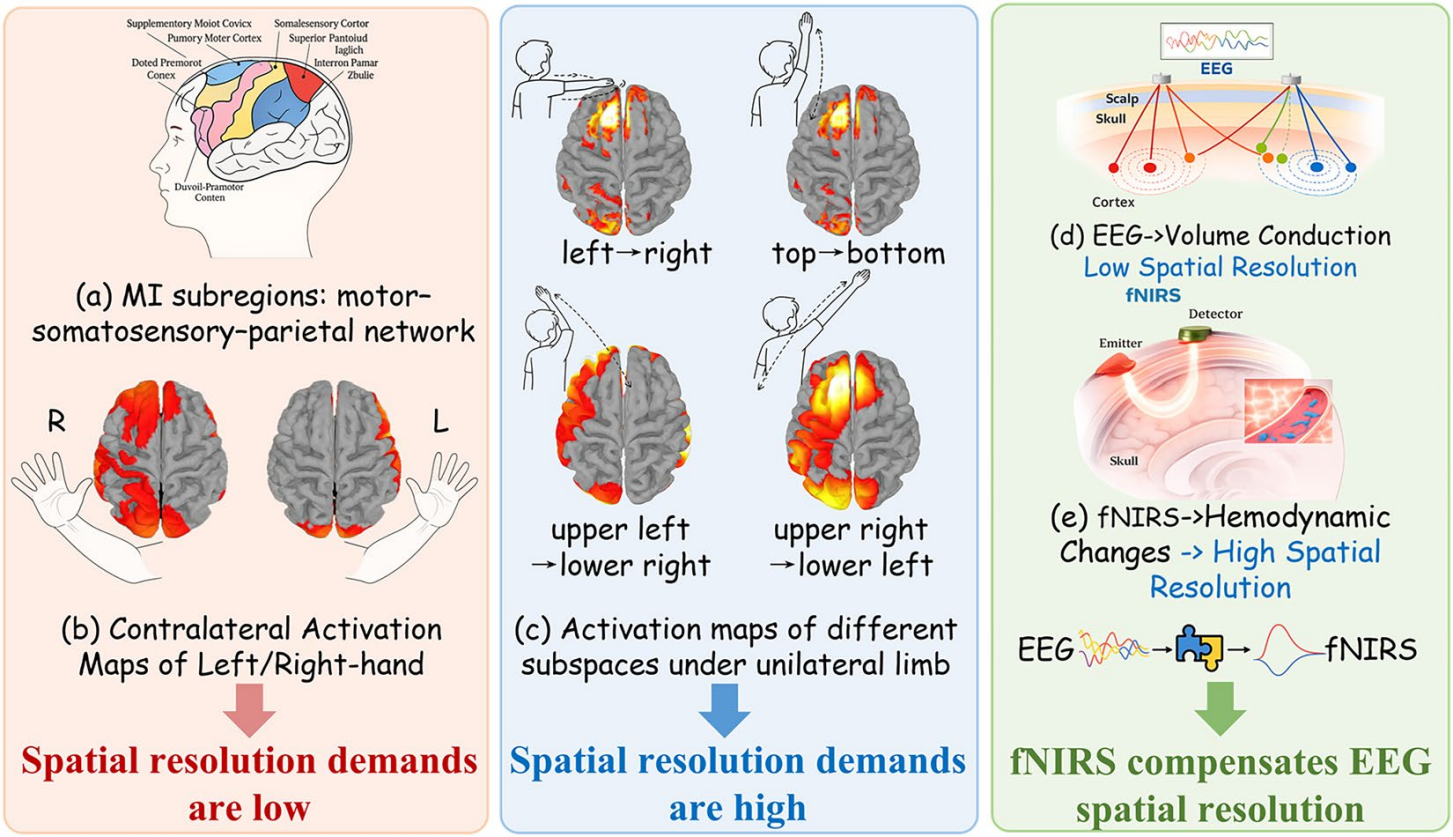
Motivation framework of the dataset. (**a**) MI subregions: motor–somatosensory–parietal network (M1/S1, PMd/PMv, SMA, and SPL/IPS). (**b**) Contralateral Activation Maps of Left/Right-hand. (**c**) Activation maps of different subspaces under unilateral limb. (**d**) Mechanism of EEG recording. Due to volume conduction, EEG has low spatial resolution. (**e**) Mechanism of fNIRS recording. fNIRS measures local ∆HbO/∆HbR with higher spatial specificity. All activation maps are plotted on a common cortical surface; brighter colors indicate stronger MI-related activity.

MI involves a distributed motor–somatosensory–parietal network, including the primary motor cortex (M1), primary somatosensory cortex (S1), lateral premotor cortex (PMd/PMv), supplementary motor area (SMA), and the superior parietal lobule / intraparietal sulcus (SPL/IPS) (Fig. 1(a))^8^. Traditional MI paradigms mainly focus on binary- or four-class bilateral limb tasks that are well separated in spatial location (e.g., left vs. right hand, feet and tongue MI)^3^. These paradigms are commonly used for algorithm development and benchmarking^9^. In these tasks, class discrimination mainly relies on hemispheric lateralization differences. As shown in Fig. 1(b), bilateral limb MI tasks often allow class discrimination by identifying which hemisphere is more active; therefore, their requirement for spatial resolution is relatively low^10^. In contrast, application scenarios such as upper-limb stroke rehabilitation^11^, unilateral exoskeleton control^12^, or prosthetic control^13^ require higher-dimensional control commands. Traditional MI paradigms provide limited classes and control degrees of freedom, and may not meet the need for fine and continuous upper-limb control.

Unilateral MI with multiple directions and multiple joints has a more intuitive correspondence to practical needs (e.g., upper-limb rehabilitation, and daily assistive control). Without substantially increasing cognitive load, unilateral MI can provide higher control freedom and better action separability^14,15^, and thus has become an active topic in MI-BCI research in recent years^16^. Unilateral multi-class MI typically does not show strong cross-hemisphere contrasts. Instead, class differences are reflected by subtle spatial modulations within the motor-somatosensory-parietal network of the same hemisphere^17^. For example, in M1/PMC/SPL, representations of the hand, wrist, elbow, and shoulder are not isolated, non-overlapping “blocks”^8^. They form a proximal-distal somatotopic gradient along the precentral gyrus^18^. Different movement directions (e.g., rightward abduction vs. right-upward lifting) often manifest as small shifts of the activation centroid within this map, and as differences in coupling with parietal regions involved in spatial attention and motor planning (e.g., SPL/IPS)^19^. Figure 1(c) illustrates local cortical differences across four unilateral tasks, suggesting that directional MI differences are finer-grained and thus impose higher demands on imaging and decoding spatial resolution.

Compared with bilateral paradigms, unilateral multi-joint and multi-direction MI depends more on high-precision spatial information. Due to volume conduction, each scalp electroencephalography (EEG) electrode (e.g., around C3, C1, and CP3) records a weighted mixture of multiple nearby cortical sources (Fig. 1(d)), leading to low spatial resolution^20^. Therefore, relying on scalp EEG alone can cause source mixing, which averages out subtle source-level differences and limits unilateral MI decoding performance. Moreover, most existing unilateral directional multi-class datasets are EEG-only. To fill this gap, we introduce functional near-infrared spectroscopy (fNIRS), which provides higher spatial specificity. fNIRS measures local hemodynamic responses via $\triangle {HbO}/\triangle {HbR}$, offering a more spatially precise description of cortical activation (Fig. 1(e)) and complementary spatial information to EEG, as supported by prior studies^21^. In addition, fNIRS has relatively good cortical spatial resolution, is more robust to electromyography (EMG) /electrical noise artefacts, and can be recorded simultaneously with EEG without mutual interference. Compared with functional magnetic resonance imaging (fMRI), fNIRS is low-cost, portable, and practical for wider use^22^. Simultaneous fNIRS–EEG acquisition therefore provides complementary spatiotemporal information for studying unilateral directional MI.

To compensate for the limited spatial resolution of EEG, we design a multimodal fNIRS–EEG unilateral upper-limb four-class directional motor imagery paradigm and release an open dataset, named Motor Imagery fNIRS–EEG Dataset (MIND)^23^. Compared with existing public datasets, the main characteristic of the present dataset does not lie in any single element alone, but in the combination of four properties: unilateral upper-limb motor imagery, four directional classes within the same limb, simultaneous fNIRS–EEG acquisition, and public release in a standardized format.

Table 1 summarizes representative MI-BCI datasets and related studies. Most paradigms use bilateral 2-class or 4-class MI tasks and are primarily based on single-modality EEG, such as BCI Competition IV^24,25^, PhysioNet EEG- MI^26^, Cho 2017^27^, OpenBMI^28^, and Kaya 2018^29^, which are widely used benchmarks for algorithm development. With the shift toward practical applications, more realistic EEG datasets have been released, including cross-day settings (e.g., Cross-Session MI^30,31^), paradigms with interference tasks (e.g., MI under Distraction^32^), and datasets designed for stroke rehabilitation (e.g., Acute Stroke MI^33^ and Lower-limb Stroke Longitudinal^34^). However, these datasets are still largely limited to bilateral paradigms or EEG-only recordings. Meanwhile, a small number of multimodal fNIRS–EEG and unilateral MI datasets have emerged. For example, Shin 2017^35^ provided a hybrid fNIRS–EEG dataset for left-/right-hand MI and mental arithmetic. OpenNeuro^36^ collected fNIRS–EEG signals during limb MI. Yi *et al*.^16^ released an fNIRS–EEG dataset for unilateral upper-limb multi-joint MI in Scientific Data. Rong *et al*.^14^ reported EEG decoding performance for unilateral upper-limb multi-class directional MI. In addition, some related unilateral multi-joint MI paradigms (e.g., Guo *et al*.^37^) have been studied but have not been released as complete public datasets. To our knowledge, publicly reusable datasets that jointly provide unilateral upper-limb tasks, four directional classes, simultaneous fNIRS–EEG recordings, and standardized public release remain scarce. MIND was constructed to address this gap.

**Table 1:** Comparison of representative MI-BCI datasets and related studies.

Dataset / Year	fNIRS–EEG	Unilateral	Directional	public	Notes
BCI-IV-2a (2008)	_**—**_	_**—**_	_**—**_	**✓**	L/R hand, foot, tongue
BBCI-IV-2b (2008)	_**—**_	_**—**_	_**—**_	**✓**	L/R hand
PhysioNet EEG-MI (2004)	_**—**_	_**—**_	_**—**_	**✓**	L/R; both fists/both feet
Cho (2017)	_**—**_	_**—**_	_**—**_	**✓**	L/R hand
OpenBMI (2019)	_**—**_	_**—**_	_**—**_	**✓**	MI/ERP/SSVEP
Kaya (2018)	_**—**_	_**—**_	_**—**_	**✓**	Up to 6 imagery tasks; includes 4-class MI settings
Cross-Session MI (2022)	_**—**_	_**—**_	_**—**_	**✓**	L/R hand
MI under Distraction (2020)	_**—**_	_**—**_	_**—**_	**✓**	Reach/MI with an interference task
Acute Stroke MI (2024)	_**—**_	**✓**	_**—**_	**✓**	L/R hand-grip imagery (stroke)
Lower-limb Stroke	_**—**_	**✓**	_**—**_	**✓**	Lower-limb gait MI (with
^Longitudinal^ (2025)	_**—**_	_**—**_	_**—**_		sensory cueing/stimulation)
TU Berlin hBCI (2017)	**✓**	_**—**_	_**—**_	**✓**	L/R + MA (mental arith metic)
OpenNeuro (2022)	**✓**	_**—**_	_**—**_	**✓**	Grasp → Twist → Reach → Lift
Guo (2024)	**✓**	_**—**_	_**—**_	_**—**_	L/R
Rong (2024)	_**—**_	**✓**	**✓**	_**—**_	Unilateral upper-limb 4-class MI (direction)
Yi (2025)	**✓**	**✓**	_**—**_	**✓**	Unilateral upper-limb multi-joint MI
Yang (2025)	_**—**_	_**—**_	_**—**_	**✓**	MI (L/R/feet)
Proposed	**✓**	**✓**	**✓**	**✓**	Unilateral upper-limb 4-class MI (direction)

**✓** indicates “yes”, and – indicates “no / not reported”.

The dataset provides raw recordings to support downstream analysis and future benchmarking. To characterize data quality and basic verifiability, we provide time-domain, frequency-domain, and topographic analyses of the simultaneously acquired EEG and fNIRS signals, together with standardized single-modality and multimodal classification summaries. These checks are included as technical validation to show that the dataset contains reproducible task-related information, rather than to establish the superiority of any specific decoding method. Recent methodological advances in related physiological-signal decoding tasks further highlight the importance of well-structured multimodal datasets for evaluating robust and reusable neural-signal analysis methods^38–44^. We expect that MIND will provide a useful open resource for multimodal MI research, spatiotemporal feature modeling, and benchmark development in both standalone EEG-based and hybrid fNIRS–EEG BCI systems.

### Methods

#### Participants

In this study, we recruited 30 healthy participants, including 18 males and 12 females, aged 19–25 years, with a mean age of 21.4 ± 1.8 years. All participants were recruited from university student volunteers to reduce variability in age and educational background. In terms of handedness, 28 participants were right-handed and 2 were ambidextrous, with no purely left-handed individuals included (Table 2). All participants had normal or corrected-to-normal vision and reported no history of neurological, psychiatric, or musculoskeletal disorders that could affect the experimental results.

**Table 2:** Demographic characteristics of the participants.

Indicators	Value
Subjects	30
Age range	19 - 25
Mean age	21.4 ± 1.8
Sex	18 males, 12 females
Handedness	28 right-handed, 2 ambidextrous
Vision	Normal or corrected-to-normal
Health status	No neurological or psychiatric disorders
Recruitment	University student volunteers

The study protocol was approved by the Ethics Committee of the Institute of Automation, Chinese Academy of Sciences (IA21-2504-020201). All procedures complied with institutional review board guidelines and relevant privacy regulations. Before the experiment, participants were given a detailed explanation of the study purpose, experimental procedures, data collection process, and data-sharing plan. All participants voluntarily signed written informed consent forms before data collection, including consent for their anonymized EEG and fNIRS data to be publicly shared and reused for non-commercial purposes under the Creative Commons Attribution-NonCommercial 4.0 International License (CC BY-NC 4.0).

### Experimental paradigm

**Fig. 2:**
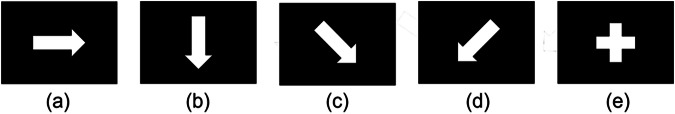
Visual instructions for each unilateral limb task. (**a**) horizontal movement from left to right. (**b**) vertical movement from top to bottom. (**c**) diagonal movement from upper left to lower right. (**d**) diagonal movement from upper right to lower left. (**e**) rest.

The experimental paradigm was designed to elicit cortical activation patterns associated with unilateral upper-limb motor imagery (MI). Four directional MI tasks were included: horizontal Left-to-Right, vertical Top-to-Bottom, diagonal Upper-left to Lower-right, and diagonal Upper-right to Lower-left (Fig. 2).

**Fig. 3:**
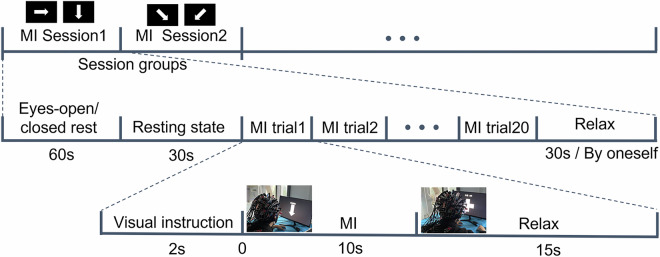
Experimental Paradigm of unilateral limb MI. Each participant completed three session groups, and each group contained two MI sessions: one horizontal/vertical session and one diagonal session, totaling six sessions. Each session included a resting state and 20 MI trials, making a total of 120 task trials.

As shown in Fig. 3, each participant completed three session groups, and each session group contained two MI sessions, resulting in six MI sessions in total. In each session group, the first MI session included the horizontal Left-to-Right and vertical Top-to-Bottom tasks, whereas the second MI session included the two diagonal tasks, namely Upper-left to Lower-right and Upper-right to Lower-left. Before each MI session, a 60 s resting period was recorded. The resting condition alternated between eyes-open and eyes-closed rest across the two MI sessions within each session group. In addition, each MI session included a 30 s resting-state preparation period before the MI trials and a 30 s self-paced relaxation period after the MI trials. These two 30 s periods were used to stabilize the participant’s state and reduce fatigue, and were not counted as MI trials or included in task-trial analysis.

Each MI session contained 20 MI trials, and each MI trial lasted 27 s, including a 2 s visual instruction period, a 10 s MI period, and a 15 s inter-trial rest period. At the beginning of each MI trial, participants received simultaneous visual and auditory cues. The visual cue consisted of a white directional arrow and the Chinese text “开始想象” (“Start imagining”) on a black background, accompanied by a short “ding” sound. During the 10 s MI period, participants were instructed to continuously imagine performing the cued limb movement and were encouraged to mentally repeat the movement 2–4 times. During resting periods, a white fixation cross was displayed on a black background. Across all six MI sessions, each participant completed 120 MI trials, with 30 trials for each direction. The trial order was randomized within each session to reduce adaptation or fatigue effects caused by a fixed sequence. Irrelevant visual or auditory stimuli were strictly avoided throughout the experiment.

Before formal data collection, all participants received detailed task instructions and completed brief MI training to become familiar with the different directional imagery tasks and the experimental procedure. During training, the researchers provided guidance to ensure that participants clearly understood the requirement to perform motor imagery only, without executing any actual movement.

During data acquisition, participants were instructed to maintain a stable seated posture, keep their bodies relaxed, and avoid overt movements of the arms, fingers, shoulders, and face as much as possible. Throughout the recording, the experimenters continuously observed and supervised participants to reduce interference from obvious actual movements in the EEG and fNIRS signals. In addition, the experimental environment was kept quiet to minimize the influence of external stimuli on participant state.

### Data collection and preprocessing

#### Data collection

**Fig. 4:**
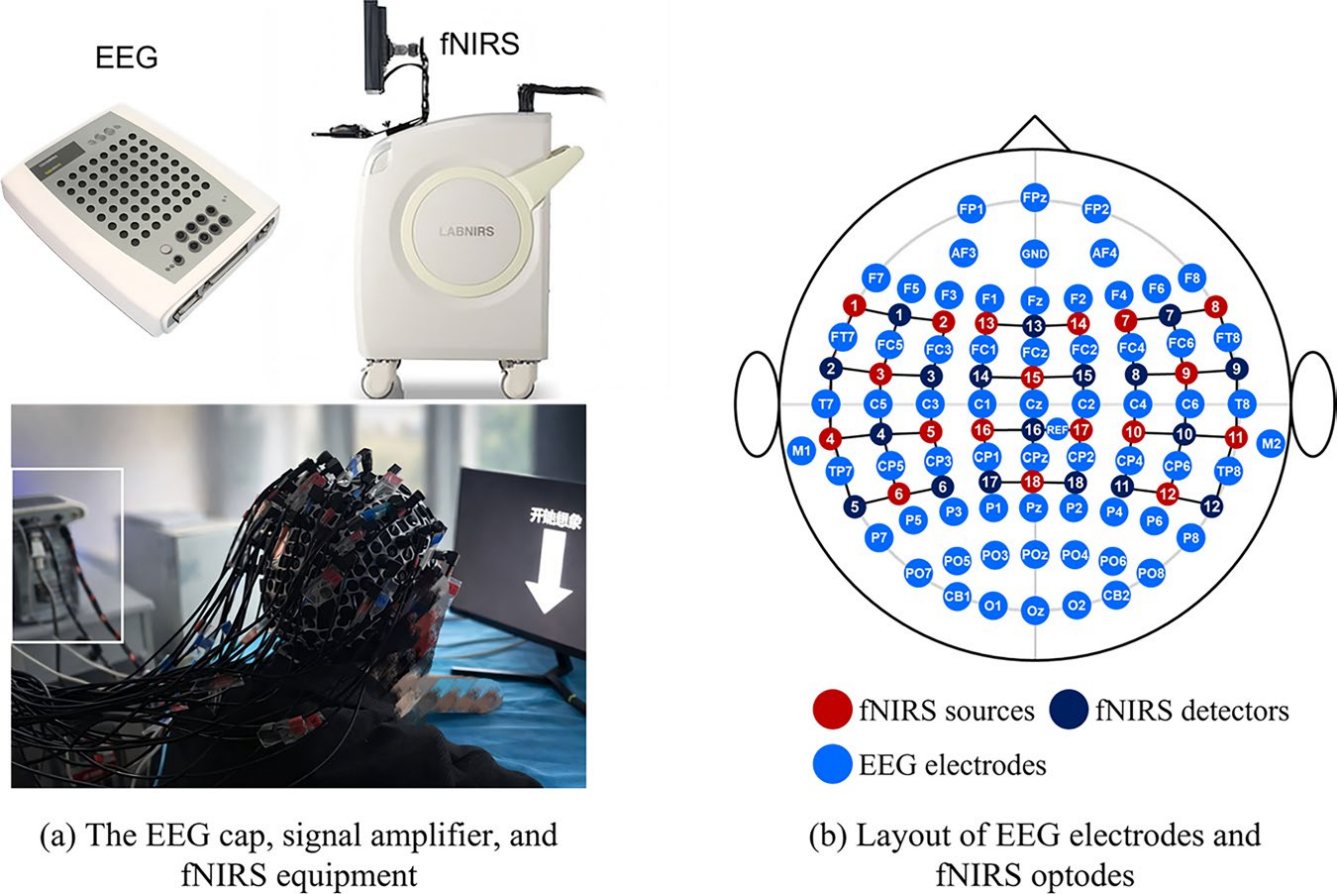
The instrumentation used in data collection. (**a**) The EEG cap, signal amplifier, and fNIRS equipment. (**b**) Layout of EEG electrodes and fNIRS optodes.

An integrated fNIRS–EEG cap and signal synchroniser developed by RSx&r was used to enable joint acquisition and precise temporal alignment of both modalities (Fig. 4(a)). The channel layout for the integrated fNIRS–EEG recording is shown in Fig. 4(b). All recordings were conducted in a controlled environment to minimise ambient light interference and movement-related artefacts, and participants were instructed to remain as still as possible throughout the experiment. The experimental paradigm was designed and implemented using E-Prime 3.0 and its development environment E-Studio^45^. Timing synchronisation between the fNIRS–EEG recording system and the experimental paradigm was achieved via digital triggers sent from E-Prime to the amplifiers, ensuring precise alignment between task events and physiological data.

EEG signals were recorded using a solid electrode cap equipped with 64 Ag/AgCl electrodes arranged according to the international 10–20 system (Neuroscan). This type of electrode cap provides stable signal quality and low impedance. Signal acquisition was performed with a Neuroscan synAmps2 wireless amplifier, which supports real-time impedance monitoring and operates in a wireless transceiver mode (Fig. 4(a)). The reference electrode (REF) was placed over the parietal region, and the ground electrode (GND) was positioned on the forehead. EEG signals were sampled at 1000 Hz. During data collection, electrode impedance was maintained below 10 kΩ to ensure signal quality. The spatial distribution of electrodes on the EEG cap is illustrated in Fig. 4(b).

fNIRS signals were recorded using a continuous-wave LABNIRS system (Shimadzu Corp., Kyoto, Japan) to measure task-related hemodynamic responses. The device operated at three wavelengths (780, 805 and 830 nm). In total, 18 emitters and 18 detectors were used, forming 51 measurement channels; the spatial layout of these channels is shown in Fig. 4(b). Before each recording, the optical pathways were adjusted and checked to ensure that the signal quality of every channel met the manufacturer’s recommended criteria. The LABNIRS system exported both device-derived hemoglobin concentration changes and three-wavelength optical absorbance signals. The standardized SNIRF files were generated from the absorbance signals, whereas the HbO and HbR concentration time series were provided as derivatives and used for technical validation and classification.

In the present study, fNIRS signals were acquired with a 51-channel continuous-wave LABNIRS system (Fig. 4(a)). The optode arrangement followed the international 10–20 system, with source–detector pairs placed mainly over the prefrontal, parietal, temporal and sensorimotor cortices to capture movement-related hemodynamic activity. The interoptode distance was set to 30 mm to ensure appropriate cortical penetration depth, and all optodes were secured using an elastic cap to maintain stable contact throughout the sessions. Data were sampled at 47.62 Hz and recorded continuously during the entire experiment.

### Data preprocessing

#### EEG preprocessing

EEG data were preprocessed using the MNE-Python toolbox^46^ together with a separate EEGLAB script. For each participant, the three raw Curry recordings were first loaded and concatenated. Event annotations were then filtered so that only resting-state and MI events with labels 1, 2, 4, 5, 6, and 7 were retained. Channels showing persistent abnormal amplitudes were marked as bad channels and excluded, after which the continuous EEG signals were band-pass filtered at $0.5-50{Hz}$ and notch-filtered at $50{Hz}$ to reduce slow drifts, high-frequency noise, and power-line interference. The bad channels were then reconstructed using spherical interpolation.

For MI data, epochs were extracted from −5 s to 20 s relative to cue onset, with baseline correction applied from −2 s to 0 s. For resting-state data, epochs were extracted from 0 to 60 s without baseline correction. All EEG epochs were subsequently resampled to 250 Hz and exported as participant-level.mat files for downstream analysis. ICA-based artifact removal was then performed in EEGLAB on the epoched EEG data. Specifically, ICA decomposition was computed using runica, with PCA reduction to 30 components. The resulting independent components were classified using ICLabel, and components were automatically rejected when the probability for muscle activity was ≥ 0.9 or the probability for eye activity was ≥0.9. Rejected components were removed using pop_subcomp. Finally, the preprocessed EEG data were exported in MATLAB-compatible.mat format for subsequent decoding and statistical analysis.

#### fNIRS preprocessing

fNIRS data were preprocessed using the MNE-Python toolbox^46^. First, the scalp coupling index (SCI) was computed from the raw device-exported multi-wavelength optical signals to assess channel quality, and channels with SCI < 0.75 were marked as bad and corrected using interpolation from neighboring channels. Next, a third-order Butterworth band-pass filter (0.02–0.2 Hz) was applied to each channel to reduce slow drift and high-frequency noise while preserving task-related hemodynamic fluctuations. Using event markers, task-related time windows (e.g., from −5 to 25 seconds around stimulus onset) were then extracted and segmented into single trials, yielding a three-dimensional data array with dimensions ${trials}\,\times \,{channel}s\times \,{time}$. A baseline interval from −5 to 0 seconds before task onset was used for baseline correction to remove slow offsets and drift. Finally, the fNIRS data were down-sampled to 10 Hz to reduce computational load while retaining physiologically meaningful information. The preprocessed HbO and HbR time series were exported in $.{csv}$ format for subsequent analysis.

## Data Records

The entire MIND dataset is publicly available on the ScienceDB platform (10.57760/sciencedb.34326)^23^. The released data are licensed for non-commercial purposes under the Creative Commons Attribution-NonCommercial 4.0 International License (CC BY-NC 4.0).

**Fig. 5:**
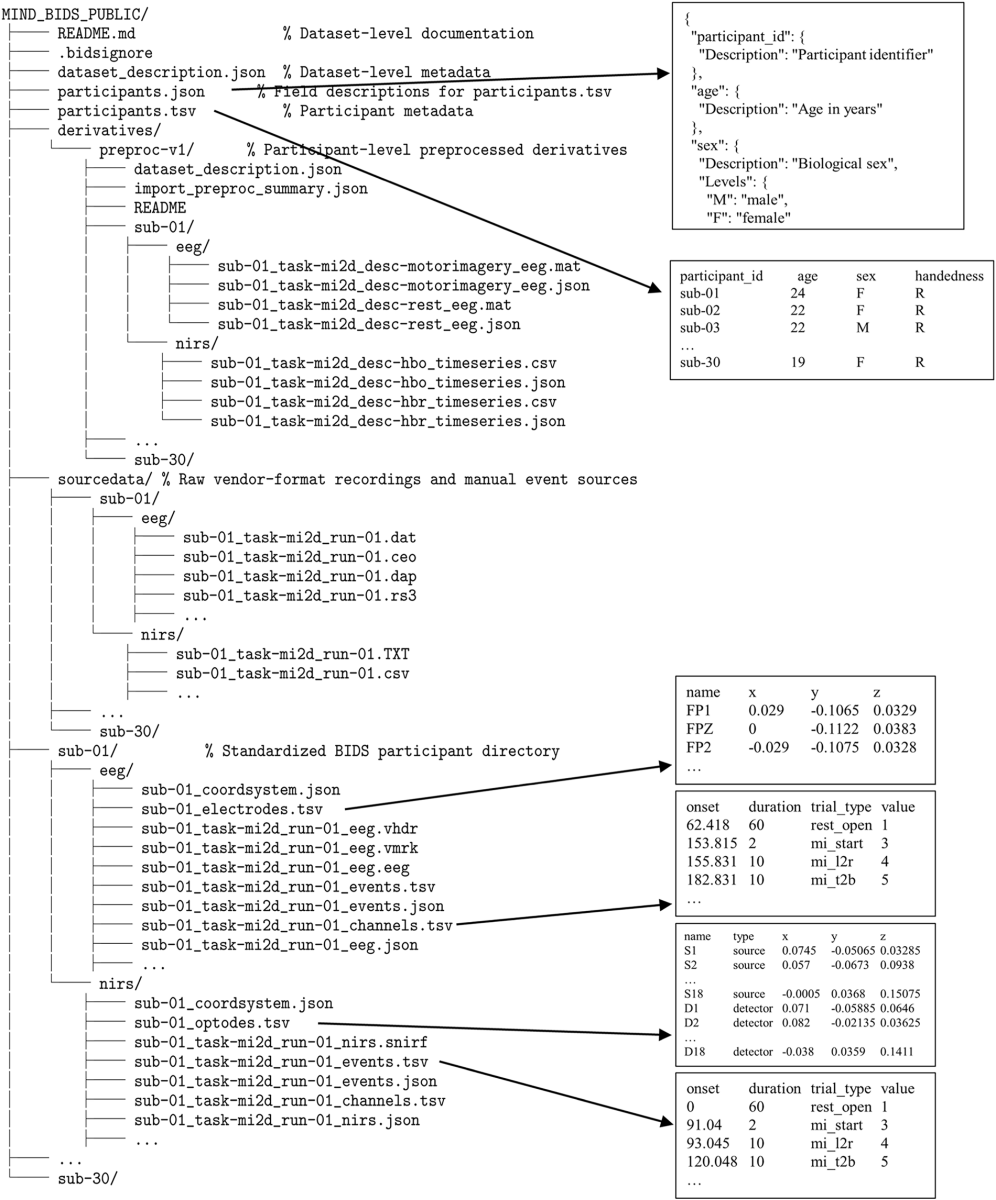
Directory structure of multimodal fNIRS–EEG dataset.

The dataset is organized according to the BIDS structure, and the data tree is illustrated in Fig. 5. The dataset root contains dataset-level description files, participant information tables, the raw vendor-format recording directory sourcedata/, standardized participant directories sub-*, and the participant-level derivative directory derivatives/preproc-v1/. The original EEG and fNIRS vendor-format files are preserved in sourcedata/, while the standardized EEG and fNIRS data are organized under the eeg/ and nirs/ subdirectories of each participant folder, respectively.

In the standardized part of the dataset, EEG recordings have been converted to BrainVision format and are accompanied by event files, channel definition files, electrode coordinate files, and coordinate system description files. For fNIRS, the original LABNIRS exports contained device-derived hemoglobin concentration changes, including oxyHb, deoxyHb, and totalHb, as well as three-wavelength optical absorbance signals, including Abs780nm, Abs805nm, and Abs830nm. In the standardized BIDS dataset, Abs780nm, Abs805nm, and Abs830nm were converted into SNIRF files to preserve information closer to the original optical measurements. The derived HbO and HbR concentration time series were not stored as the primary data in the SNIRF files, but were released as derivative.csv files in derivatives/preproc-v1/. The fNIRS data are accompanied by event files, channel definition files, source and detector position tables, and coordinate system description files. Preprocessed results are provided as derivatives in derivatives/preproc-v1/, including participant-level EEG.mat files and fNIRS HbO/HbR.csv time-series files with corresponding description files.

The dataset includes two special acquisition cases. First, the second fNIRS acquisition of sub-07 was interrupted during recording and was therefore split into two separate segments, acq-a and acq-b, which were preserved separately in the standardized directories. Second, the second fNIRS acquisition of sub-13 was corrupted. These cases have been explicitly documented in the data structure and metadata to ensure consistency and transparency between the released data and the original acquisition process.

## Technical Validation

### fNIRS–EEG synchronization

To characterize the synchronization consistency between EEG and fNIRS recordings, we separately analyzed the automatic event-marker group and the manually corrected group. The automatic event-marker group included 19 participants and was used for cross-modal event matching and relative temporal consistency analysis. The manually corrected group included 11 participants and was used only to examine event counts, label sequences, and annotation traceability, and was not included in the temporal residual statistics.

Because EEG and fNIRS recordings may have different recording start times, the first common event within each MI session was used as the temporal reference to calculate the relative time difference:

1 $${\triangle }_{i}={(t}_{i}^{{fNIRS}}-{t}_{1}^{{fNIRS}})-{(t}_{i}^{{EEG}}-{t}_{1}^{{EEG}}),$$

where ${t}_{i}^{{fNIRS}}$ and ${t}_{i}^{{EEG}}$ denote the time of the $i-{th}$ common event in the fNIRS and EEG files, respectively, ${t}_{1}^{{fNIRS}}$ and ${t}_{1}^{{EEG}}$ denote the time of the first common event within the session. We further used a linear model to fit the relationship between the event time axes of the two modalities:

2 $${t}^{{fNIRS}}=a+b{t}^{{EEG}}+\varepsilon ,$$

where a represents the constant offset between the two file time axes, b represents the relative time-axis scaling factor, and ε represents the residual after linear correction. Drift was defined as $\left(b-1\right)\times 60\times 1000\,{ms}/\min$. For statistical analysis, metrics were first calculated for each participant and then summarized at the group level.

The 19 participants with automatic event markers yielded 114 complete MI sessions and 2394 common events. The EEG and fNIRS event counts, event codes, and event sequences were consistent across all sessions, and no unmatched experimental-design events were found, indicating complete event correspondence between the two modalities. The group-level relative time difference was 9.43 ± 62.90 ms, with a 95% CI including zero, indicating no consistent directional systematic temporal offset. The median drift was −0.51 ms/min, and the median residual SD after linear correction was 5.09 ms (Table 3). Since the fNIRS sampling rate was 47.62 Hz, corresponding to a sampling interval of approximately 21.0 ms, the median residual SD and maximum absolute residual of typical sessions were below one fNIRS sampling interval. These results indicate that the EEG and fNIRS event markers in most released recordings showed stable relative temporal consistency. Nevertheless, individual sessions may still contain relatively large discrete event offsets. Therefore, for studies requiring millisecond-level cross-modal temporal relationships, we recommend independently checking the event time axes of the target sessions before analysis.

**Table 3:** Synchronization consistency statistics of fNIRS–EEG events in the automatic event-marker group (n = 19).

Run-Session	Relative time difference, mean ± SD (ms)	95% CI (ms)	Drift, median [IQR] (ms/min)	Residual SD, median [IQR] (ms)
Run01-S1	1.48 ± 151.76	−71.67 to 74.62	−0.35 [−1.23, 0.03]	5.74 [4.78, 6.60]
Run01-S2	37.90 ± 172.63	−45.30 to 121.11	−0.54 [−1.77, −0.04]	5.44 [3.62, 7.46]
Run02-S1	26.87 ± 153.15	−46.94 to 100.69	−0.63 [−1.56, −0.11]	6.06 [4.15, 8.39]
Run02-S2	−16.97 ± 9.93	−21.76 to −12.19	−0.41 [−0.75, −0.11]	4.97 [3.77, 6.54]
Run03-S1	−45.95 ± 96.98	−92.70 to 0.79	−0.63 [−4.08, −0.13]	5.02 [4.40, 6.61]
Run03-S2	53.28 ± 232.31	−58.69 to 165.25	−0.35 [−1.66, −0.03]	4.82 [4.02, 6.92]
Average	9.43 ± 62.90	−20.88 to 39.75	−0.51 [−0.91, −0.11]	5.09 [4.23, 5.82]

Note: Relative time differences were calculated from participant-wise session means and then summarized at the group level. For the pooled result, six sessions were first averaged within each participant and then summarized across participants. SD denotes standard deviation, and IQR denotes interquartile range.

**Fig. 6:**
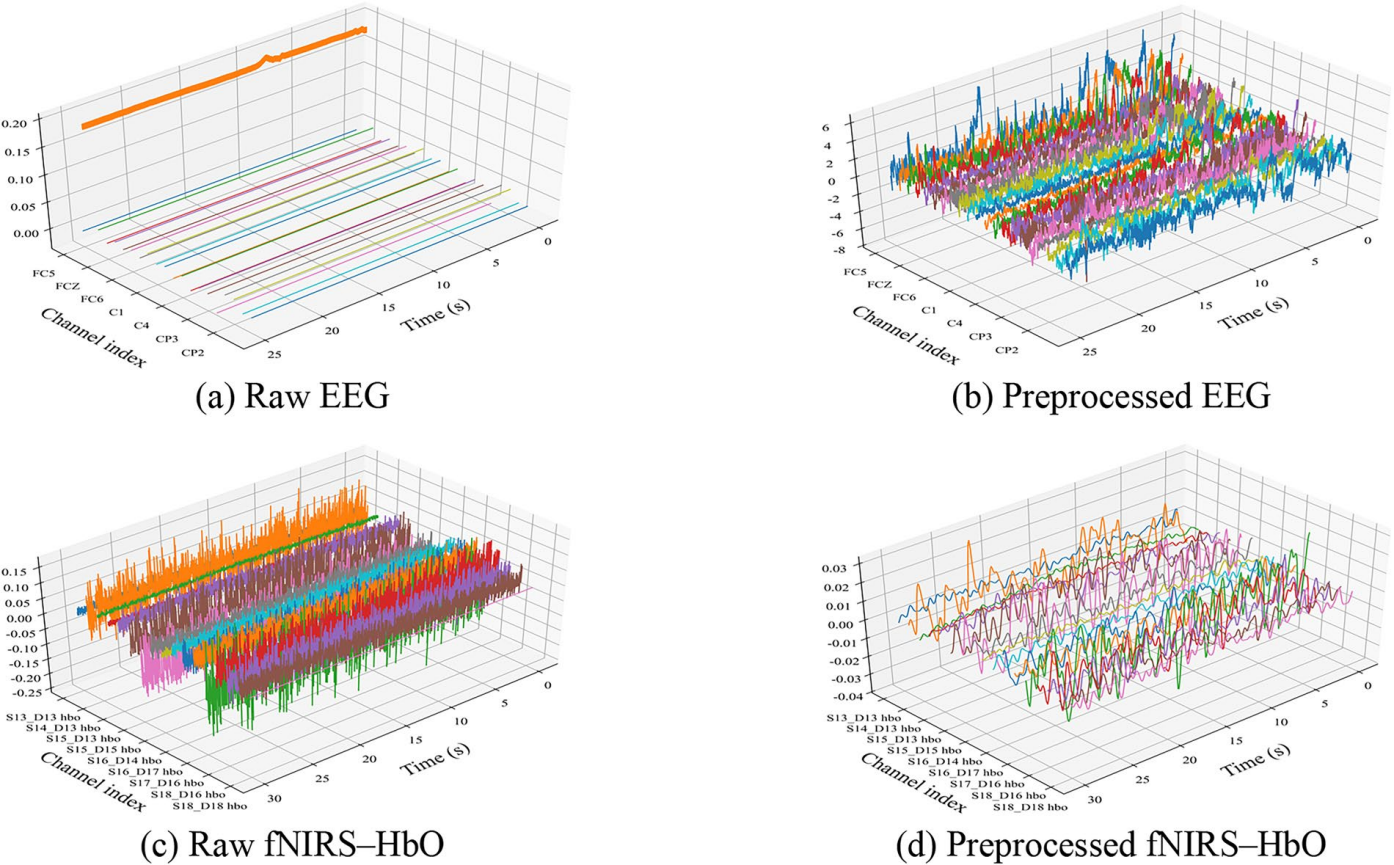
Three-dimensional visualizations of multichannel raw and preprocessed EEG and fNIRS signals. (**a**) Raw EEG; (**b**) Preprocessed EEG; (**c**) Raw fNIRS–HbO; (**d**) Preprocessed fNIRS–HbO.

To facilitate intuitive channel-level assessment of data quality and the identification of potentially abnormal channels, three-dimensional visualizations of multichannel EEG and fNIRS signals are provided in Fig. 6. The figure presents comparisons between raw and preprocessed signals, where time is shown on the horizontal axis, channels are arranged along one spatial dimension, and signal amplitude is represented as the third dimension.

From the EEG visualizations, it can be observed that some channels in the raw data exhibit obvious abnormal amplitude fluctuations and transient spikes, appearing as trajectories that deviate markedly from the other channels. Such patterns are typically associated with poor electrode contact or transient artifacts. After bad-channel identification, neighboring-channel interpolation, and routine preprocessing, these abnormal fluctuations are effectively suppressed, and the overall signals show improved consistency and stability across channels.

For the fNIRS signals, some channels in the raw HbO data exhibit amplitude offsets and enhanced high-frequency noise, appearing in the three-dimensional view as abnormal trajectories that clearly deviate from the overall channel distribution. After signal-quality-based channel screening and interpolation, these abnormal channels are corrected, and the signals become smoother overall while showing a more physiologically plausible temporal pattern.

### EEG data analysis

#### Time–domain analysis (ERD/ERS)

To validate EEG signal quality at the dataset level, we performed group-level ERD/ERS analysis across 30 participants. Event-related desynchronization/synchronization (ERD/ERS) was used to quantify changes in sensorimotor rhythms closely related to MI, focusing on the $\alpha /{mu}$ (8–12 Hz)^47^. The left C3 ROI included FC3, C3, CP3, FC5, C5, and CP5, while the right C4 ROI included FC4, C4, CP4, FC6, C6, and CP6. The computation proceeded as follows: (1) the preprocessed EEG signals were band-pass filtered in the α band; (2) the filtered signals were squared at each sample point to obtain instantaneous power; (3) for each trial, the mean power during the −2 to 0 s pre-cue interval was used as the baseline; (4) power time series were averaged across channels within each ROI to obtain the left and right sensorimotor ROI power time courses; and (5) trials were first averaged within each task for each participant, followed by grand averaging across the 30 participants. ERD/ERS was expressed as:

3 $$\frac{{ERD}}{{ERS}\left(t\right)}=\,\frac{P\left(t\right)-{P}_{{baseline}}}{{P}_{{baseline}}}\times 100 \% ,$$

where $P\left(t\right)\,$ denotes the task-related power at time t, and ${P}_{{baseline}}\,$ denotes the baseline power. Negative values indicate power decreases relative to baseline, corresponding to ERD; positive values indicate power increases relative to baseline, corresponding to ERS.

**Fig. 7:**
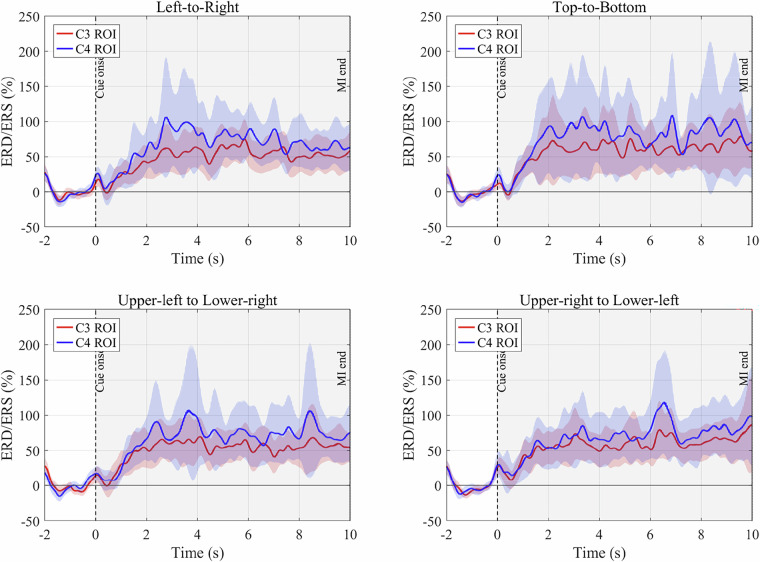
Grand-average EEG ERD/ERS in the α band (8–12 Hz) across 30 participants. Shaded areas indicate 95% confidence intervals. Baseline was −2 to 0 s. C3 ROI included FC3, C3, CP3, FC5, C5, and CP5; C4 ROI included FC4, C4, CP4, FC6, C6, and CP6.

The group-average ERD/ERS curves for the four MI tasks are shown in Fig. 7. All curves were computed based on 30 participants, and the shaded areas indicate 95% confidence intervals. Overall, both the C3 ROI and C4 ROI showed task-locked rhythmic changes, with the average change in the C4 ROI generally being larger than that in the C3 ROI. During the 0–10 s task period, the mean ERD/ERS was 53.3 ± 60.3% for the C3 ROI and 69.3 ± 100.9% for the C4 ROI. For example, in the Left-to-Right task, the mean ERD/ERS was 48.2 ± 54.8% for the C3 ROI and 66.3 ± 88.4% for the C4 ROI, with a C4–C3 difference of 18.0% (95% CI: 0.01 to 36.05%, p = 0.0499). These results indicate clear task-locked mu rhythm modulation at the group level. Since the group-average curves generally showed positive power changes, the results suggest that right-hand directional motor imagery induced sensorimotor rhythm modulation involving both ERD and ERS components, with inter-subject variability. The lower amplitude in the C3 ROI compared with the C4 ROI may reflect relatively stronger desynchronization components in the contralateral left sensorimotor region.

#### Frequency-domain analysis (ERSP)

Event–related spectral perturbation (ERSP) was used to characterise changes in spectral energy during MI^48^. ERSP is an EEG analysis method that examines changes in oscillatory power across time and frequency. Compared with ERD/ERS time courses, ERSP can simultaneously show power increases or decreases at different frequency components relative to baseline. In this study, group-level ERSP was computed using the short-time Fourier transform (STFT). The procedure was as follows: (1) STFT decomposition was applied to the EEG signals for each participant, task, trial, and ROI; (2) the spectral power at each time-frequency point was computed as:

4 $${Power}(f,t)={|{F}(f,t)|}^{2},$$

where F(f,t) denotes the complex STFT value at frequency f and time t; (3) trials from the same task and ROI were averaged within each participant; and (4) the −2 to 0 s interval was used as the baseline, and dB normalization was performed for each frequency:

5 $${ERSP}\left(f,t\right)=10{{\log }}_{10}\frac{P\left(f,t\right)}{{P}_{{baseline}}\left(f\right)},$$

where $P(f,{t})$ denotes the task-related time-frequency power, and ${P}_{{baseline}}(f)$ denotes the average power of the corresponding frequency during the baseline interval. Negative values indicate power decreases relative to baseline, corresponding to ERD, whereas positive values indicate power increases relative to baseline, corresponding to ERS. The subject-level ERSP results were then grand-averaged across all participants.

**Fig. 8:**
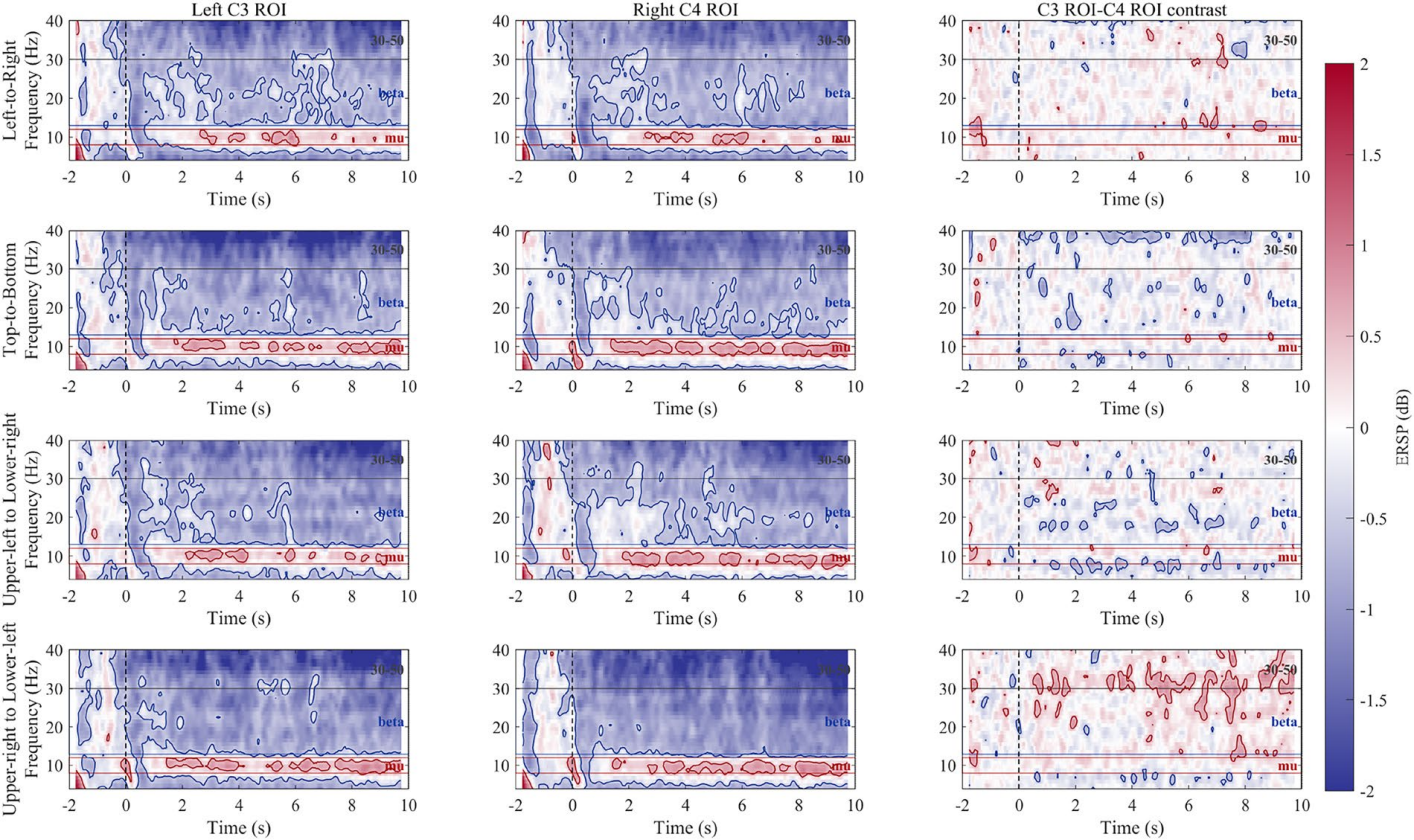
Group-average ERSP maps over left and right sensorimotor areas during four MI tasks. ERSP was normalized in dB relative to the −2 to 0 s baseline. Left C3 ROI included FC3, C3, CP3, FC5, C5, and CP5; right C4 ROI included FC4, C4, CP4, FC6, C6, and CP6.

The group-average ERSP distributions for the four MI tasks are shown in Fig. 8. The first and second columns show the ERSP results for the C3 ROI and C4 ROI, respectively, and the third column shows the C3 ROI minus C4 ROI contrast. The frequency range was 4–40 Hz, and the time range was −2 to 10 s. Overall, both the C3 ROI and C4 ROI showed clear frequency-specific modulation after cue onset. Relative power increases were observed around the 8–12 Hz mu band, whereas sustained power decreases were observed in the 13–30 Hz beta band and the 30–40 Hz higher-frequency range. During the 0–10 s interval, the mu-band ERSP was 0.29 ± 1.02 dB in the C3 ROI and 0.29 ± 1.22 dB in the C4 ROI. The beta-band ERSP was −0.75 ± 1.03 dB and −0.74 ± 1.07 dB in the C3 and C4 ROIs, respectively, both below baseline. The 30–40 Hz frequency range also showed relative decreases, with values of −1.54 ± 1.73 dB and −1.43 ± 1.91 dB in the C3 and C4 ROIs, respectively. The C3 ROI minus C4 ROI contrast showed similar overall time-frequency patterns between the two ROIs, indicating that the EEG motor-imagery effect in this dataset showed stable sensorimotor rhythm modulation, but without pronounced lateralization.

**Fig. 9:**
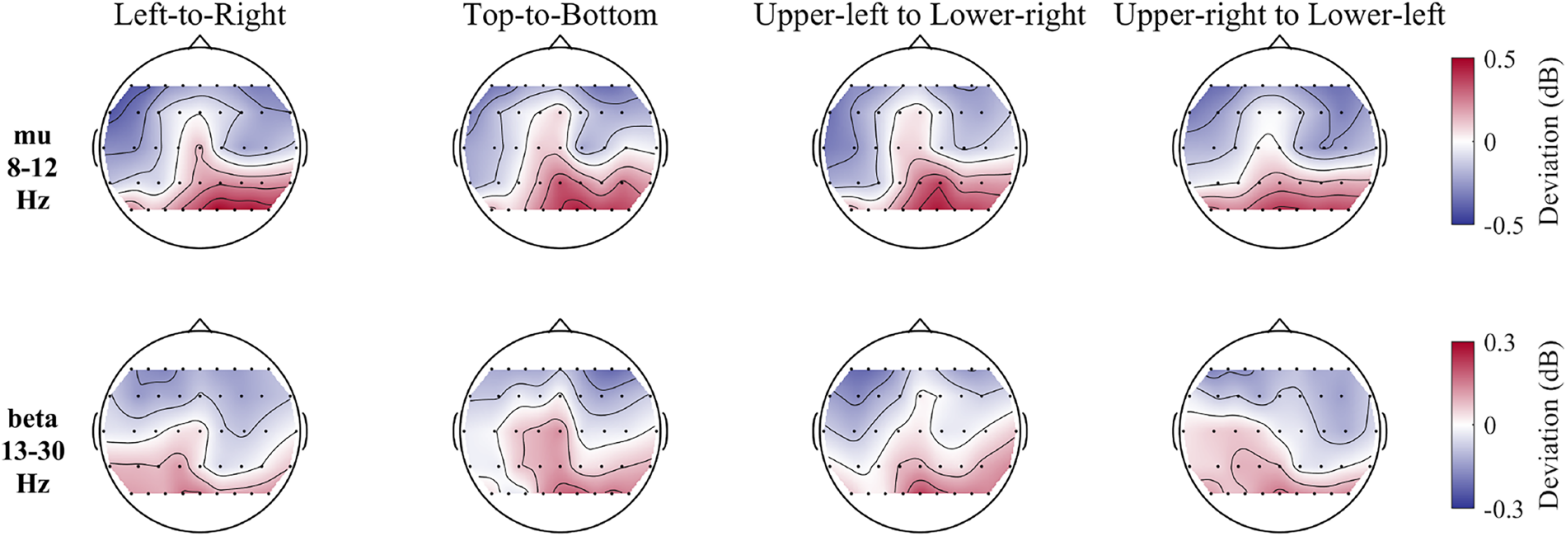
Group-average EEG spatial deviation maps of baseline-normalized mu- and beta-band power changes during the 0–10 s motor imagery period. The baseline window was −2 to 0 s, and values are expressed in dB after removing the whole-head mean.

The EEG spatial distributions are shown in Fig. 9. This figure presents the group-average spatial deviation maps across 30 participants, which were used to reflect the relative spatial differences in task-related spectral changes across brain regions. Specifically, for each participant, task-period band power changes were first computed relative to baseline, and the whole-head mean was then removed to highlight the degree of local deviation relative to the overall scalp distribution. The figure uses the 45 electrodes actually covering the frontal, central, and parietal regions, without excessive interpolation over regions without electrode coverage. As shown in the figure, the four tasks exhibited relatively consistent frontal–central–parietal spatial gradients in both the mu and beta bands. The spatial deviation ranged approximately from −0.42 to 0.51 dB in the mu band and from −0.23 to 0.23 dB in the beta band. Slight spatial differences were observed between the left and right sensorimotor ROIs, with minor variations across tasks, but no strong lateralization was observed.

### fNIRS data analysis

Since right-hand motor imagery is typically associated with activity in the contralateral left sensorimotor region, we first selected a left-hemisphere fNIRS ROI for the HbO and HbR time-course analysis. The left-hemisphere ROI consisted of 17 channels formed by source-detector pairs from sources 1–6 and detectors 1–6. Each trial was baseline-corrected using the −5 to 0 s pre-cue interval. The signals were then averaged across channels within the ROI and across trials of the same task within each participant to obtain subject-level HbO and HbR responses. Finally, the subject-level results from 30 participants were grand-averaged.

**Fig. 10:**
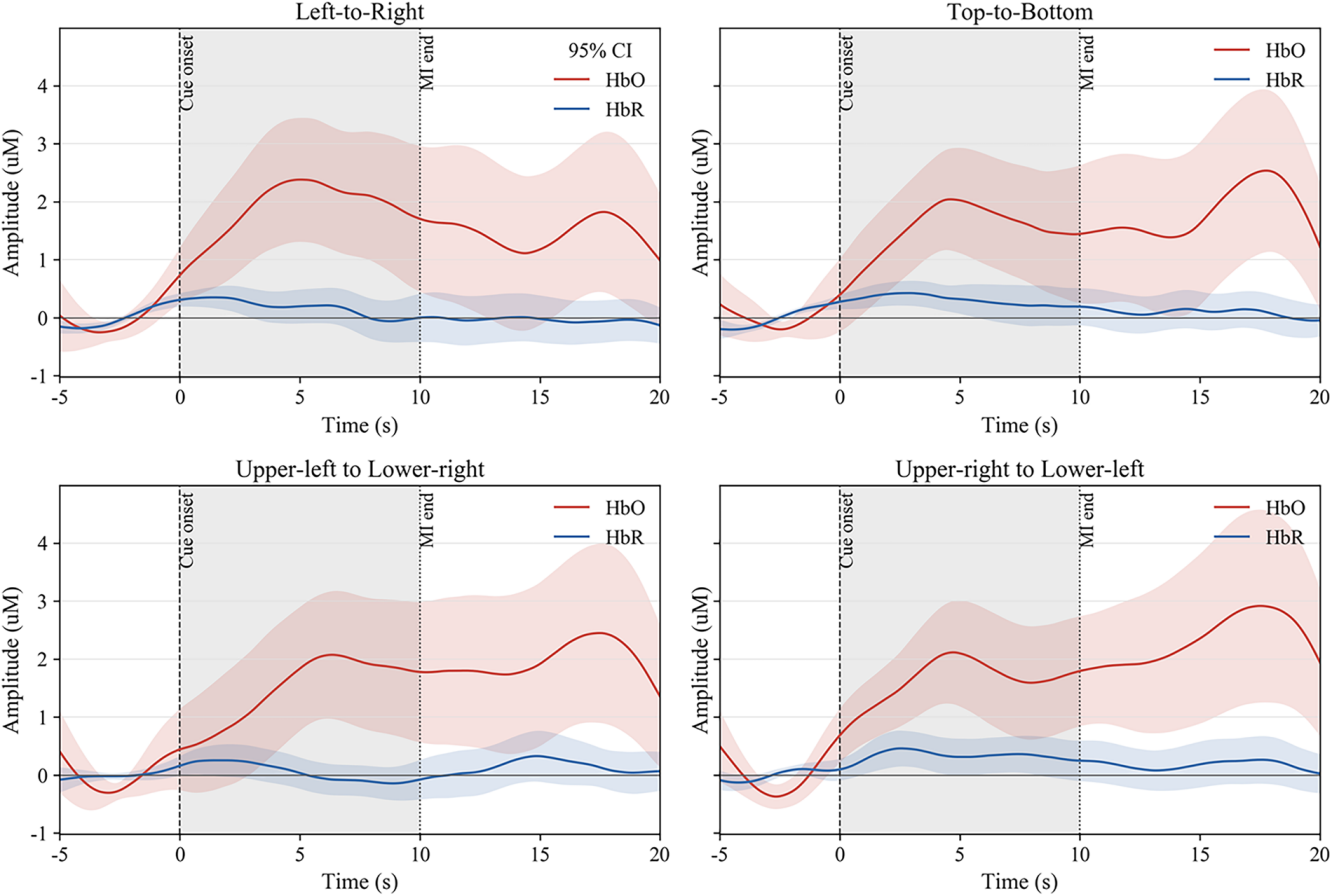
Group-average fNIRS HbO/HbR responses over left-hemisphere ROI for four directional MI tasks across 30 participants. Shaded areas indicate 95% confidence intervals. The left-hemisphere ROI consisted of 17 channels formed by source-detector pairs from sources 1–6 and detectors 1–6.

The group-average HbO and HbR responses for the four MI tasks are shown in Fig. 10. The x-axis represents time from −5 to 20 s, and the y-axis represents concentration changes in μM. Across all four tasks, HbO showed a stable increase after cue onset, whereas HbR changes were smaller and remained close to baseline. During the 0–10 s interval, the mean HbO change was 1.62 ± 1.79 μM, with a 95% CI of 0.96 to 2.29 μM and P < 0.0001. During the 4–10 s interval, HbO further increased to 1.89 ± 2.26 μM, with a 95% CI of 1.05 to 2.74 μM and P < 0.0001. By contrast, HbR was 0.21 ± 0.55 μM during 0–10 s and 0.15 ± 0.66 μM during 4–10 s, indicating much smaller changes than HbO. The HbO responses generally increased several seconds after cue onset and, in some tasks, persisted beyond task offset, consistent with the delayed nature of fNIRS hemodynamic responses relative to neural activity.

**Fig. 11:**
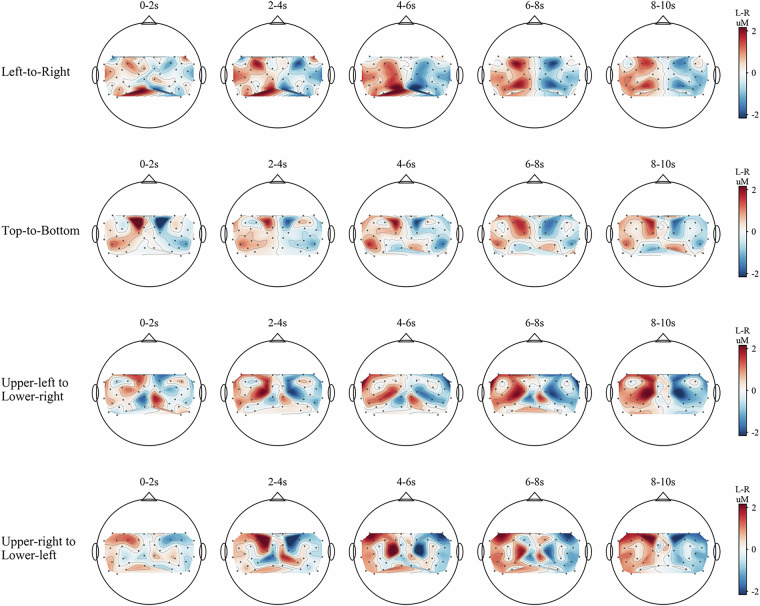
Group-average fNIRS HbO lateralization maps during four directional MI tasks. Maps show left-minus-right HbO responses from mirrored channel pairs after −5 to 0 s baseline correction.

The HbO left–right hemispheric difference topography is shown in Fig. 11. The maps were computed from mirrored channel pairs as the left-hemisphere HbO response minus the right-hemisphere HbO response, with positive values indicating stronger HbO responses in the left hemisphere. Since all tasks involved right-hand motor imagery, the left hemisphere was contralateral to the imagined movement; thus, positive L–R differences may reflect enhanced contralateral hemodynamic responses. Each trial was baseline-corrected using the −5 to 0 s interval, after which the mean HbO change was calculated within each time window and then averaged at the subject and group levels. During 0–10 s, the mean HbO L–R difference across all tasks was 0.51 ± 1.04 μM, with a 95% CI of 0.12 to 0.90 μM and P = 0.0115. During 4–10 s, the difference was 0.61 ± 1.21 μM, with a 95% CI of 0.15 to 1.06 μM and P = 0.0103. Although the spatial patterns varied across tasks, the overall results showed stronger HbO responses in the left hemisphere than in the right hemisphere. This finding is consistent with the expected contralateral hemodynamic enhancement during right-hand MI and provides additional group-level evidence for the fNIRS data quality of the dataset.

### fNIRS–EEG classification

To perform a basic check on whether stable task-related information is present in the data, we adopted a unified sliding-window classification framework to evaluate EEG, fNIRS, and their combinations. All classification analyses were conducted in MATLAB R2019b using the BBCI toolbox^49^. For EEG, with cue onset defined as time zero, sliding windows of 3 s length and 1 s step were applied from −5 s to 20 s. Within each window, spatial features were extracted using Filter Bank Common Spatial Pattern (FBCSP)^50^, and the log-variance of 36 CSP components was computed. The classifier was shrinkage regularized linear discriminant analysis (sLDA), implemented with a one-versus-rest strategy for four-class classification.

For fNIRS, the same sliding-window setting was used. Within each window, the mean concentration and slope of HbO and HbR were extracted from each channel as features. For multimodal checking, the sLDA projection scores from single-modality classifiers were combined to form meta-features, and four combinations were constructed: HbR + HbO, EEG + HbR, EEG + HbO, and EEG + HbR + HbO. All results were obtained under subject-dependent evaluation. Within each subject, the evaluation was conducted using 5-fold cross-validation, where the data were split into training (80%) and test (20%) sets in each fold. Spatial filters were estimated only from the training set and then applied to the corresponding test set to avoid information leakage.

**Fig. 12:**
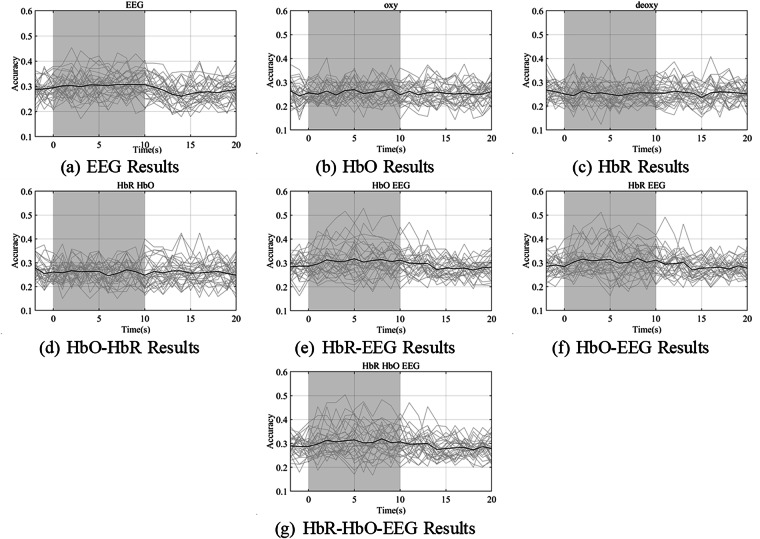
EEG, fNIRS, and fNIRS + EEG classification accuracies for 3 s moving time window for MI. The x-axis indicates the right edge of the moving time window. Gray lines show the individual classification accuracies while thick black lines show the across-subject average accuracies. The task starts at 0 s and finishes at 10 s. Gray shaded areas indicate task periods.

Based on this unified framework, we summarized the four-class results using the theoretical chance level of 25% as a reference (Fig. 12 and Table 4). For the window-averaged results, the mean accuracy across the seven modality configurations ranged from 26.41% to 31.93%, with standard deviations from 3.75% to 7.56%. The corresponding 95% confidence intervals ranged from 24.99% to 34.25%. Using the theoretical chance level of 25% as a reference, we summarize the four-class results under a subject-dependent baseline evaluation. Across modality configurations, the mean accuracies were above chance and the reported p-values were below 0.05. These results indicate that the data contain reproducible task-related information at the overall window level. Based on the same framework, we further summarized the best-window four-class results using the theoretical chance level of 25% as a reference (Fig. 12 and Table 5). For the best-window results, the mean accuracy across the seven modality configurations ranged from 31.63% to 37.53%, with standard deviations from 2.93% to 6.50%. The corresponding 95% confidence intervals ranged from 30.41% to 39.79%, all above the chance level. These results indicate that the dataset contains reproducible task-related information and support its usability from a data quality perspective. These results are presented as baseline reference analyses for future data reuse and should not be interpreted as evidence that the current fusion strategy is optimal. The EEG baseline performance was comparable in magnitude to that reported in a previous related study^14^, providing an external reference for interpreting the baseline validation results (FBCSP: 32.71 ± 13.19).

**Table 4:** All window-averaged four-class classification accuracy across all subjects: classification accuracy mean (Mean), standard deviation (SD), 95% confidence interval (CI), and p-value against the chance level (0.25).

Modality	Mean(%)	SD(%)	95% CI	P vs. 0.25
EEG	30.95	4.83	[29.11, 32.79]	p = 7.60e-06
HbO	27.14	4.04	[25.60, 28.67]	p = 5.68e-03
HbR	26.41	3.75	[24.99, 27.84]	p = 1.91e-02
HbO + HbR	27.08	4.91	[25.21, 28.95]	p = 2.39e-02
EEG + HbO	31.78	7.56	[28.91, 34.66]	p = 9.95e-05
EEG + HbR	31.81	6.15	[29.47, 34.15]	p = 1.43e-05
EEG + HbO + HbR	31.93	6.12	[29.60, 34.25]	p = 8.78e-06

**Table 5:** Best window-averaged four-class classification accuracy across all subjects: mean, standard deviation (SD), 95% confidence interval (CI), and p-value against the chance level (0.25).

Modality	Optimal window	Mean(%)	SD(%)	95% CI	P vs. 0.25
EEG	8	36.19%	3.97%	[34.68, 37.70]	p = 1.27e-06
HbO	9	31.94%	3.44%	[30.63, 33.25]	p = 1.27e-06
HbR	2	31.63%	3.19%	[30.41, 32.84]	p = 1.28e-06
HbO + HbR	8	33.39%	2.93%	[32.28, 34.50]	p = 1.25e-06
EEG + HbO	5	37.13%	6.50%	[34.65, 39.60]	p = 1.27e-06
EEG + HbR	8	37.01%	5.69%	[34.85, 39.18]	p = 1.27e-06
EEG + HbO + HbR	8	37.53%	5.94%	[35.27, 39.79]	p = 1.27e-06

When reusing this dataset, users should consider that the participants were mainly healthy young university students, which may limit generalization to broader or clinical populations. The reported decoding results were obtained using a simple sliding-window FBCSP + sLDA baseline without cross-subject validation, and should therefore be treated as conservative reference results rather than upper-bound performance. In addition, we performed a subject-level check of the fusion results. Among 29 (excluding sub-13) comparable subjects, EEG + HbO showed the largest number of improvements over EEG at windows 2 and 7, both with 17 out of 29 subjects. EEG + HbR showed the largest improvement at window 2, with 18 out of 29 subjects; for the three-modality fusion, 17 out of 29 subjects achieved performance not lower than EEG at window 2.

## Data Availability

The usage instructions for this dataset are openly available in our GitHub mirror repository (https://github.com/useflf/Multimodal-fNIRS-EEG-Dataset). The folder Code/Preprocessing/ contains preprocessing scripts for both EEG and fNIRS. For EEG preprocessing, /EEG_process.py and EEG preprocessing /EEGPreprocess.m are provided. For fNIRS preprocessing, /2024_11 _11_snirf_trans_merge.ipynb is used for automatic event labeling, and fNIRS preprocessing /no mrk.ipynb is used for manual label refinement. The folder Code/Plot/ includes scripts for EEG time–frequency analysis and topographic mapping, as well as fNIRS averaged hemodynamic response analysis and topographic visualization. The folder Code/Classification/ provides unimodal and multimodal classification code based on the FBCSP + sLDA pipeline. All researchers are free to download, use, and cite these resources. We recommend reading the accompanying documentation carefully before using the dataset, and following the original acquisition protocol and ethics statement.
